# Improving the Precision of Deep-Learning-Based Head and Neck Target Auto-Segmentation by Leveraging Radiology Reports Using a Large Language Model

**DOI:** 10.3390/cancers17121935

**Published:** 2025-06-10

**Authors:** Libing Zhu, Jean-Claude M. Rwigema, Xue Feng, Bilaal Ansari, Jingwei Duan, Yi Rong, Quan Chen

**Affiliations:** 1 Department of Radiation Oncology, Mayo Clinic, Phoenix, AZ 85058, USA; zhu.libing@mayo.edu (L.Z.); rwigema.jean@mayo.edu (J.-C.M.R.); ansaribmo34@lakeforest.edu (B.A.); 2 Carina Medical LLC., Lexington, KY 40513, USA; xfeng@carinaai.com; 3 Department of Physics, Lake Forest College, Lake Forest, IL 60045, USA; 4 Department of Radiation Oncology, The University of Alabama at Birmingham, Birmingham, AL 35233, USA; jingweiduan@uabmc.edu

**Keywords:** auto-segmentation, GTV, head and neck, large language model, clinical diagnosis report

## Abstract

**Background/Objectives**: The accurate delineation of primary tumors (GTVp) and metastatic lymph nodes (GTVn) in head and neck (HN) cancers is essential for effective radiation treatment planning, yet remains a challenging and laborious task. This study aims to develop a deep-learning-based auto-segmentation (DLAS) model trained on external datasets with false-positive elimination using clinical diagnosis reports. **Methods**: The DLAS model was trained on a multi-institutional public dataset with 882 cases. Forty-four institutional cases were randomly selected as the external testing dataset. DLAS-generated GTVp and GTVn were validated against clinical diagnosis reports to identify false-positive and false-negative segmentation errors using two large language models: ChatGPT-4 and Llama-3. False-positive ruling out was conducted by matching the centroids of AI-generated contours with the slice locations or anatomical regions described in the reports. Performance was evaluated using the Dice similarity coefficient (DSC), 95th percentile Hausdorff distance (HD95), and tumor detection precision. **Results**: ChatGPT-4 outperformed Llama-3 in accurately extracting tumor locations from the diagnostic reports. False-positive contours were identified in 15 out of 44 cases. The DSC_mean_ of the DLAS contours for GTVp and GTVn increased from 0.68 to 0.75 and from 0.69 to 0.75, respectively, after the ruling-out process. Notably, the average HD95 value for GTVn decreased from 18.81 mm to 5.2 mm. Post ruling out, the model achieved 100% precision for GTVp and GTVn when compared with the results of physician-determined contours. **Conclusions**: The false-positive ruling-out approach based on diagnostic reports effectively enhances the precision of DLAS in the HN region. The model accurately identifies the tumor location and detects all false-negative errors.

## Simple Summary

Auto-segmentation of gross tumor volumes in the head and neck regions remains a challenging task. This study introduces a novel approach to eliminate the incorrect contours of primary gross tumors and metastatic lymph nodes in the head and neck region generated by the deep-learning-based auto-segmentation model, leveraging clinical diagnosis reports. By analyzing these clinical reports with a large language model, the method can accurately identify false-positive tumor contours and ensure that only genuine tumor regions are segmented. This innovative technique improves the precision of automatic tumor delineation.

## 1. Introduction

Head and neck (HN) cancer ranks among the most lethal cancers globally [1]. Radiation therapy (RT) is a widely used treatment approach for HN cancer. Accurate tumor and lymph node (LN) GTV segmentation is a critical step for ensuring the precise RT targeting of cancerous cells while minimizing damage to the surrounding healthy tissues. Fluorodeoxyglucose (FDG) positron emission tomography (PET) and computed tomography (CT) imaging are commonly used modalities for the primary and LN gross tumor volume (GTVp and GTVn) segmentation, staging, and follow-up of HN cancer [2]. FDG-PET provides metabolic information, while CT offers anatomical details, which makes them complementary for cancerous lesion segmentation. However, due to the positron annihilation ranges, PET images often appear blurred, especially near airway regions. Long travel distances of positrons through air can cause the PET signal to “bleed” into the airway. Consequently, the manual segmentation of GTVp and GTVn in the HN region is a demanding process for radiation oncologists [3], with challenges due to interobserver variability [4,5]. Significant efforts have been reported in ensuring contouring reproducibility and reducing variability to achieve optimal HN patient treatment outcomes [6,7,8].

Recent advancements in deep learning (DL)-based auto-segmentation (DLAS) offer promising improvements in contour consistency and error detection from manual contouring [9,10,11], and reduction of manual workload [12]. Professional societies and the industry have organized various grand challenges to promote and evaluate different AI tools for auto-segmentation from in-house or industry developers [13,14,15]. The first head and neck tumor segmentation challenge (HECKTOR, 2020), focusing on the automatic segmentation of HN primary tumors in PET/CT scans, achieved an aggregated dice similarity coefficient (DSC) of 0.759 for the top-performing champion [16]. The subsequent HECKTOR 2022 added a task of HN nodal gross tumor volumes segmentation and achieved aggregated DSC values of 0.788 and 0.776 for GTVp and GTVn, respectively, for the top-performing champion [9]. However, a caveat of these two grand challenges is that the real-world performance of the trained model using the challenge-provided data remains unclear.

Clinical generalization of DLAS models remains a significant challenge, as model performance often declines when evaluated on local clinical data [17,18]. Thus, local validation of deep learning technologies is crucial [17]. Moreover, the evaluation metrics (aggregated DSC) used for the HECKTOR challenge may not be clinically meaningful [19]. The aggregated DSC is calculated based on the overall volume overlap across all cases, which may undervalue errors in small tumors, despite their potentially high clinical impact. Moreover, DSC equally weights over-segmentation (false-positive) and under-segmentation (false-negative), despite their distinct clinical implications.

Tumor classification and segmentation have mostly been performed manually by radiation oncologists (RadOncs) who have received years of training and accumulated extensive experience. Nevertheless, it can still be a challenging task for humans, despite the great advancements in imaging modalities, including CT, magnetic resonance imaging (MRI), and PET. For example, when using FDG-PET, tumor cells are highlighted through FDG uptake due to their higher metabolic rates compared to non-cancerous cells, but some conditions, such as inflammations or benign tumors like granulomas, may also show increased FDG uptake [20], leading to false positives. In clinical practice, RadOncs typically rely on radiologists’ reports to delineate primary tumors and LNs. If DLAS models could utilize radiologists’ interpretation of PET-CT, which is already part of the patient’s medical record, they could potentially enhance the accuracy of tumor identification.

However, the lack of a standardized format for radiologists’ reports makes it difficult to integrate and extract key information from them, challenging the development of any automated approach [21]. Recent advancements in large language models (LLMs) offer the ability to interpret, extract, and summarize relevant medical details from free-text, human-language reports such as radiology reports [22,23,24,25,26]. LLMs have been employed to detect errors in radiology reports and to interpret complex radiological jargon for patients. By leveraging this capability, DLAS for HN tumor and LN segmentation may be able to achieve greater precision, driving its clinical adoption [27]. The performance of commercial LLMs, including proprietary and open-source ones, specifically for extracting HN GTVp and GTVn information from patients’ diagnosis reports, has not been explored. The specific aims of this study include (1) to evaluate the performance of deep learning-based HN primary tumor and involved nodal segmentation trained on the HECKTOR dataset and tested on a local clinical dataset; (2) to develop and validate a novel approach that integrates existing radiologists’ imaging reports from the patient’s medical record, in order to enhance DLAS accuracy and reliability.

## 2. Materials and Methods

### 2.1. Deep-Learning-Based Auto-Segmentation Model

#### 2.1.1. Dataset

The training dataset of the model was from the 2022 HECKTOR challenge [14], organized by Medical Image Computing and Computer Assisted Intervention (MICCAI). The dataset includes 882 cases from 9 different institutions. Each case contains FDG-PET and CT scans of the HN region and clinically approved contours for primary and nodal GTVs. The DLAS model was tested using our local dataset of 44 HN cancer cases, each consisting of separately contoured GTVp (*n* = 40) and GTVn (*n* = 111), manually drawn by RadOncs based on the planning CT images co-registered with the PET images. The planning CT/PET co-registration was performed using a commercial deformable registration workflow (version 7.2.7, MIM Software Inc., Cleveland, OH, USA), and CT/PET fusion was used as the network input.

#### 2.1.2. Network Structure and Training

A 3D U-Net network was employed with five encoding and five decoding layers, allowing for a multi-modality input with the same resolution in PET, MR, and CT. The deep supervision technique was employed to facilitate the training [28], computing the loss at each decoding block except for the bottleneck layer and the first decoding block. The loss function is a sum of cross-entropy and Dice loss. A novel channel dropout approach was utilized to emulate different input combinations of the imaging modalities, increasing the model robustness and ensuring that the network learned intrinsic features from each modality or any combination [29]. Instead of combining CT and PET images as 4D input, we randomly dropped the image from one modality by replacing it with zero arrays. The detailed network architecture can be found in the literature [30].

A patch-based approach was applied to extract small patches from each subject due to the large GPU memory demand of the input images. The training was performed on NVIDIA Tesla V100 SXM2 GPU with 16 Gb memory. The trained model achieved performance comparable to that obtained in the HECKTOR 2022 challenge [29].

### 2.2. LLM Prompting for Analyzing Clinical Diagnosis Reports

The clinical diagnosis reports of 44 cases were downloaded from the electronic medical record (EMR) platform, with all patient information anonymized. An example of the report is shown in Figure S1a. The report consists of two main paragraphs: the first is “Findings” which elaborates on all suspicious tumors identified by the radiologist, including location, standardized uptake value (SUV), origin, and histology; and the second is “Impression” which further elaborates on the types of tumors mentioned in the first paragraph. ChatGPT-4 was initially employed to extract tumor information from the reports and determine if the tumor was primary or nodal. The GPT application programming interface (API) was employed to analyze the clinical diagnostic reports for the 44 cases in batch mode with no patient or physician information included. Additionally, all the clinical reports were tested using a local LLM, large language model meta AI with 8 billion parameters (Llama-3-8B) [31,32], which was downloaded and run on a local server.

Extra information was provided to the LLM to improve its performance, a process called “prompt engineering”. We first started with a basic prompt (prompt1), i.e., “Generate a table for HN, extract slice number, tumor size (if none, leave empty), SUV value, tumor anatomic region, tumor type, whether the tumor is primary or a lymph node tumor, if multiple tumors, list each tumor at different rows. For primary tumors, the anatomic region is nasopharyngeal, oropharynx, hypopharynx, tongue base, and carotid space; extract detailed left, right, or bilateral tumors. For lymph nodes, provide the detailed lymph node level as anatomic region”. We observed that the LLMs sometimes flagged benign abnormalities as tumors, even when the report implied otherwise. An example in Figure S1b,c shows GPT misclassifying a suspected goiter as a tumor. We found that our radiologists normally summarize the major non-benign findings in the “Impressions” paragraph. Therefore, for the 2nd prompt (prompt2), we added “Use the impression paragraph to help determine if the tumor in the findings paragraph is primary or a lymph node tumor”. Prompt2 can perform more accurately than prompt1 in extracting primary tumors and LNs. Both LLMs were tested using prompt2, and the results were compared for the accuracy of information extraction for primary tumors and LNs to the RadOnc’s manual contours.

### 2.3. Workflow to Rule Out False-Positive Errors in DLAS Contours

Since there is no defined format for the radiology report of the PET-CT findings, practice varies between radiologists on how much detail is provided regarding the location of tumors and lymph nodes. The level of details can even differ within the same report. Radiologists at our institution use various methods to distinguish lesions. Often, the slice number for high-SUV regions was specified, as well as laterality, anatomical locations, sizes, and SUV. Sometimes, the slice number was omitted, and only laterality and anatomical region were reported for the lymph nodes. We propose two workflows to automatically match the contours with the lesions classified in radiology reports based on the description of the location. One is based on slice location. The other involves creating approximate masks for various anatomical regions based on the tumor’s relative location with respect to organs and structures segmented by a commercial auto-segmentation software (INTContour, Carina Medical LLC.).

#### 2.3.1. Scenario 1: Detailed Slice Location-Based Workflow

**Figure 1:**
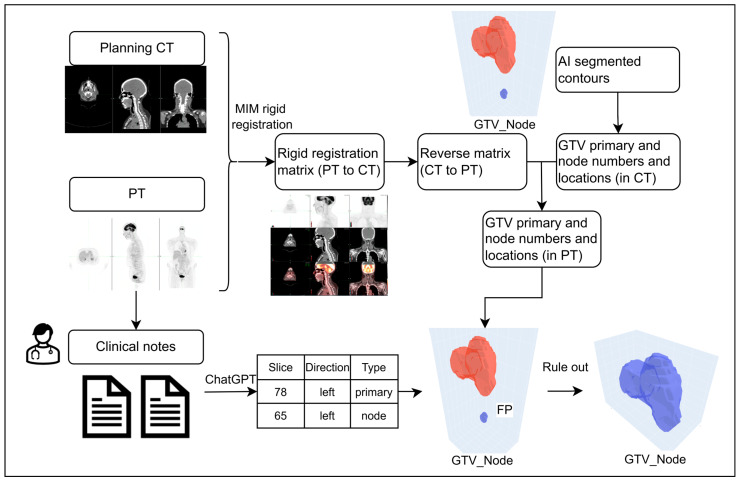
Workflow for ruling out FP contours with detailed tumor location from the diagnosis report.

The workflow for scenario 1 is shown in Figure 1, where the diagnostic report provides a detailed description of each primary tumor and lymph node. For each case, the planning CT and PET images were rigidly registered in MIM, a necessary step for contouring. The rigid registration matrix (PET to CT) was exported and converted to an inverse registration matrix. The centroids of the DLAS contours for the primary tumor and lymph nodes were identified by locating the 3D connected regions. Since the centroids of each tumor were in the CT coordinate system and the clinical diagnosis reports were in the PET coordinate system, the centroids were then converted into the PET coordinate system using the inverse registration matrix.

The DLAS contour was matched with the lesions described in the diagnosis reports in three steps: first, extract the laterality of each tumor (left or right) from the reports and compare it with the laterality of the centroid of the DLAS contours relative to the midline; second, find the centroids closest to the reported slice location; third, confirm that the DLAS contour cover the reported slice. A match was found only when all these conditions were met. Otherwise, a DLAS contour was labeled as a false positive (FP) if no corresponding reported lesion was identified. Likewise, if a tumor or LN was described in the diagnostic report but failed to be segmented, it was labelled as a false negative (FN).

#### 2.3.2. Scenario 2: Anatomic Region-Based Workflow

**Figure 2:**
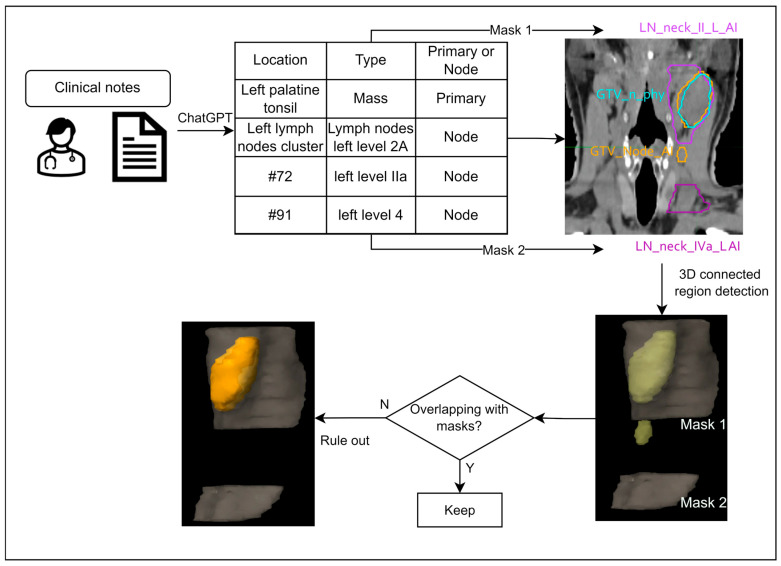
Workflow for ruling out FP contours with GTVn LN group description in diagnosis report.

Scenario 2 occurs when the radiologist does not provide detailed slice locations for each tumor and instead specifies the LN group and anatomic location of primary tumors. The workflow for LN false-positive errors ruling out is illustrated in Figure 2. The commercial software INTContour generated DLAS on the CT images, which included 50 HN organs at risk, as well as 8 LN group levels (1b to 4a). Based on the LN level information from the diagnosis reports, only the DLAS contour with its centroid located in the correct LN group was identified as a match.

**Figure 3:**
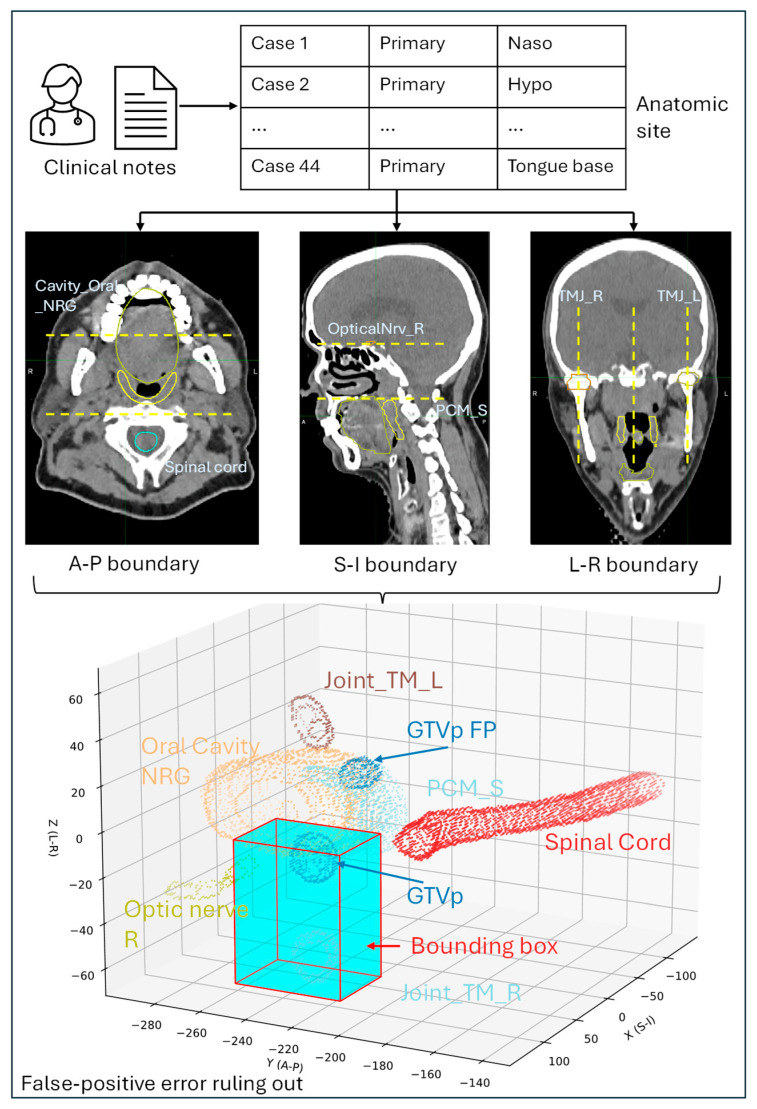
Bounding box creation from the anatomic region in the diagnosis reports. (Blue cube indicates the bounding box generated from anatomic descriptions provided in diagnostic reports).

For primary tumors, radiologists typically mention the anatomical position of the tumors in their diagnostic reports. For example, “there is a large hypermetabolic mass lesion centered at the right nasopharyngeal region and crossing the midline”. By analyzing the anatomical locations of the primary tumors in all 44 cases, the following positions were recorded: nasopharyngeal, oropharyngeal (including tongue base and oral cavity), hypopharyngeal, carotid space, and tongue base (the most common site in our analysis). A bounding box was generated for each site using the closest superior (S), inferior (I), left (L), right (R), anterior (A), and posterior (P) OARs. Figure 3 shows a detailed workflow for creating a bounding box for the right nasopharyngeal region and ruling out false-positive DLAS contours.

We developed a comprehensive anatomic position-based workflow to rule out the false-positive errors. The S-I, A-P, and L-R boundaries of the bounding boxes are summarized in Table S1 for five anatomic regions, i.e., nasopharyngeal, oropharynx, hypopharynx, tongue base, and carotid space.

### 2.4. Evaluation

The DLAS contours were initially compared to the manually delineated contours by RadOncs using the Dice similarity coefficient (DSC) and 95th percentile Hausdorff distance (HD95). Each DLAS was then reviewed against the radiologist’s report. The ruling-out approach described in Section 2.3 was applied, and the DSC, HD95, FP, and FN were updated accordingly. A Student’s *t*-test was performed to compare the DSC and HD95 values before and after the ruling-out process. A *p*-value less than 0.05 was considered statistically significant.

## 3. Results

### 3.1. Baseline Performance of the GTV DLAS Model

**Figure 4:**
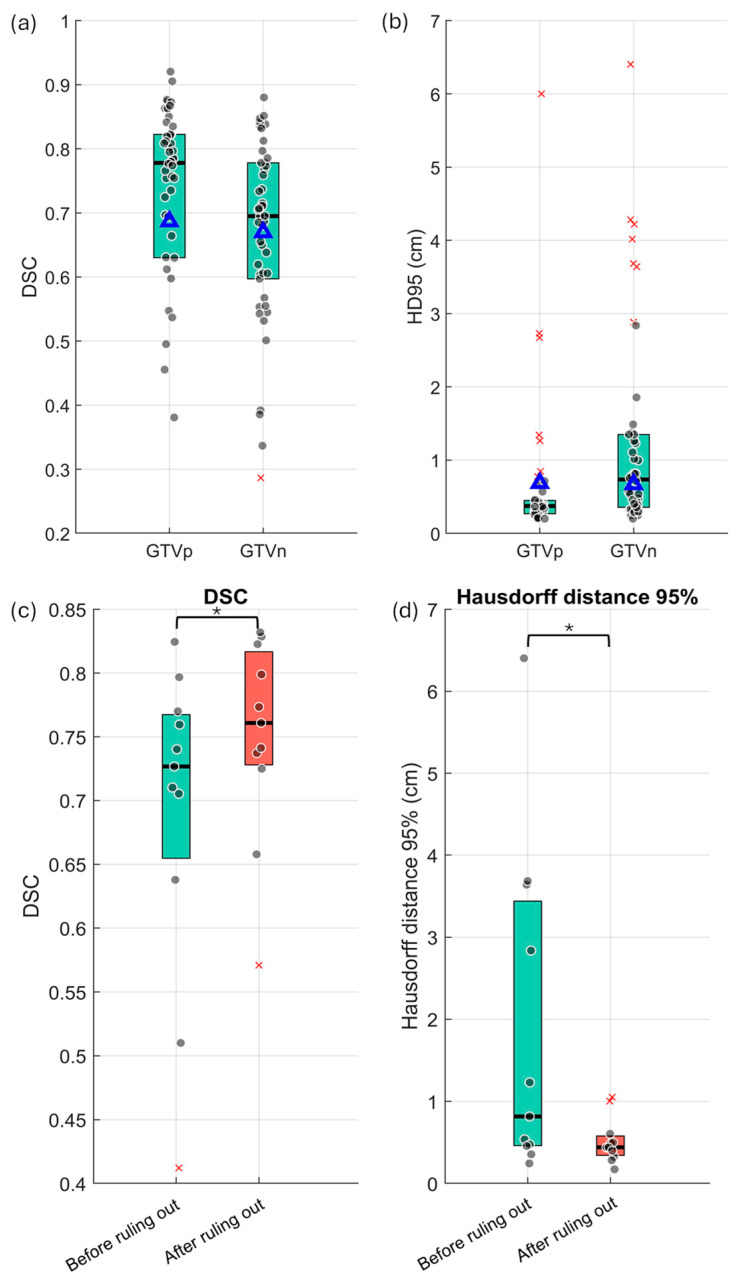
Auto-segmentation results of 44 cases for GTVp and GTVn. (**a**) DSC (**b**) Hausdorff distance 95th percentile, mean value is represented by Δ, and outliers by ×. Box ranges are 25th and 75th percentile. Comparison of DLAS contours (GTVn) before and after the ruling-out process in terms of DSC (**c**) and HD95 (**d**) for false-positive cases (*: *p*-value < 0.05).

**Figure 5:**
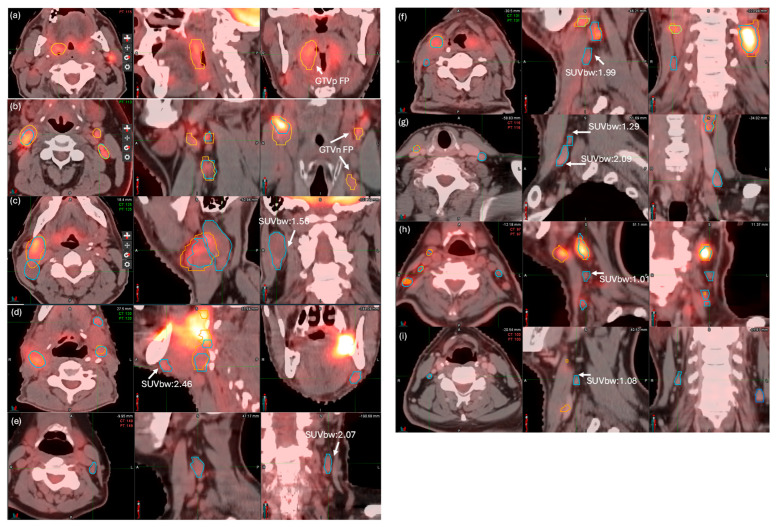
Example cases of false-positive errors for GTVp (**a**) and GTVn (**b**) and false negative errors for GTVn with small SUV value (**c**–**i**) (golden contour indicates DLAS nodes, light blue indicates RadOnc manual contour).

Figure 4a,b shows the performance of the DLAS model in the segmentation of primary tumors and lymph nodes using DSC and HD95. For GTVp, the DLAS model achieved an average DSC of 0.68 ± 0.22 and an HD95 of 5.2 ± 2.9 mm on our local data. In four cases with no primary tumor present, the DLAS erroneously generated contours, yielding a DSC of 0 and substantially lowering the overall DSC. For GTVn, the DLAS model obtained a DSC of 0.66 ± 0.17 and an HD95 of 16.0 ± 16.0 mm. Two examples of edge cases with low DSC scores and false-positive errors are shown in Figure 5a,b. Notably, both cases demonstrated that AI under-segmented the GTVn due to the low standardized uptake value normalized by body weight (SUVbw). Out of 44 cases, 5 and 12 cases contained FP errors for GTVp and GTVn, respectively. Only eight cases contained FN errors for GTVn, while no FN errors were observed for GTVp. Thus, the main cause of outliers and errors in DLAS was attributed to FP errors.

### 3.2. LLM Extraction Results

We tested GPT-4 with two prompts as described in Section 2.2. While the total RadOnc contours identified 40 GTVp and 111 GTVn, there were 47 primary tumors and 117 LNs using prompt1, and 40 primary tumors and 106 LNs using prompt2. The results of prompt2 matched better those of the RadOncs. In prompt2, 9 cases out of 44 were described with “clusters” and “several nodes”. The radiologists provided the detailed location of the tumor for the remaining 35 cases, and they were consistent with the RadOnc manual contours. It was noted that the manual GTVn contours grouped multiple nodes into single contours for nine cases, contributing to a smaller number of nodes compared with the GPT extraction. Additionally, for four cases, the RadOnc did not contour some LNs that had small SUVbw (ranging from 2.2 to 3.6), which were mentioned in the radiology report.

Llama-3-8B showed 48 primary tumors and 127 LNs. For the primary tumors, Llama-3-8B failed to merge extended regions of the same primary tumor into a single entity, which caused the number of FP primary tumors to increase. For the LNs, it extracted the LNs from the non-HN region and considered indeterminate nodes as nodes, which led to the extra FP nodes.

### 3.3. FP Ruling Out and FN Detection Results

After applying the ruling-out process, both DSC and HD95 significantly improved for 11 cases, as shown in Figure 4c,d. In one case, the clinical contour for GTVn was missed; thus, the DSC or HD95 could not be calculated. For the remaining cases, both the 25th and the 75th percentiles increased for the DSC and decreased notably for the HD, indicating better performance. The mean DSC increased from 0.69 to 0.75, while the average HD dropped from 18.81 mm to 5.20 mm after the ruling-out process. This indicates that our proposed ruling-out approach increased the DSC by 8.7% and reduced the HD by 13.61 mm. Notably, in the case with the lowest DSC value (0.41) before the ruling-out process, the score improved to 0.57 after removing the false-positive lymph node contours. These improvements were statistically significant for both DSC and HD95, with p-values of 0.011 for the DSC and 0.044 for HD95.

Table 1 provides a precision analysis of AI’s performance for the 44 cases, highlighting instances of over-segmentation (false-positive errors) and under-segmentation (false-negative error). The ruling-out process successfully removed all FPs for both GTVp (5) and GTVn (17), achieving a perfect precision of 1.0. However, eight FN errors remained for 111 GTVn instances. Figure 5c–i illustrate these eight FN error cases. As shown in the figure, all FN contours correspond to regions with very low uptakes. The standardized uptake value normalized by body weight (SUVbw) statistics for those eight FNs is reported in Table S2. The average SUVbw was only 1.55 for the eight FN errors, ranging from 0.97 to 2.09.

## 4. Discussion

Although the feasibility of DLAS for primary and lymph node target volume has been demonstrated in the HECKTOR challenge setting, its real-world performance has not been evaluated. In our study, we observed significant performance degradation for GTVp and GTVn in independent clinical cases, mainly due to the high incidence of FPs and FNs in DLAS. The interpretation of PET-CT images for primary and nodal targets can be a challenging problem for AI. Inflammation and infections may elevate the FDG uptake, leading to FPs. On the other hand, suspicious enlarged lymph nodes, even in the absence of obvious uptake, may be selected for treatment. One study reported an elevated risk of recurrence when elective radiotherapy was chosen and PET-negative lymph nodes were not treated [33], indicating the need of including PET-negative nodes in the nodal tumor volume. In our practice, the RadOnc consults the radiology report for target determination. In this study, we developed an LLM-based report reader to extract the location information for more precisely identifying tumors and nodes from the radiology report. Two approaches for automatically ruling out FP AI contours were also developed to accommodate location detail variations. The 100% success rate of our FP ruling out demonstrates the advantage of leveraging radiology reports with the LLM.

Our approach still left eight FN GTVn contours that the DLAS failed to contour. A close inspection of those eight FNs showed that they had very low SUV, as shown in Table S2. One possible explanation is that the HECKTOR challenge contains only GTVn contours with high SUV. In that case, the DLAS model was only trained by the association of GTVn with elevated SUV value. Another possible reason is that it is rare to contour PET-negative nodes as GTVn. In our dataset, this occurred in only 8/111 instances. Such an imbalanced dataset can cause DLAS models to predominantly learn from the majority samples [34], thus associating GTVn with high SUV. To address this issue, the PET-negative nodes need to be identified from the HECKTOR challenge dataset for handling imbalanced datasets [35,36]. Unfortunately, such data were not provided by the challenge organizer. In addition, the decision to include a PET-negative node as GTVn could come from the biopsy result or be subject to physician’s preference. Future work will be devoted to improving the DLAS for PET-negative nodes.

We employed the state-of-the-art LLM models GPT-4 and Llama-3 to parse the radiology report. Our observation was that both general-purpose LLM models performed well in understanding diverse medical terminologies and categorizing findings as primary vs. nodal, malignant vs. benign, etc. However, we also observed notable errors when using these LLMs directly out of the box. For example, in one instance, the report mentioned “symmetric enhancement in the tonsil region”, which GPT-4 incorrectly categorized as malignant primary tumor. Interestingly, upon prompting “When a high-uptake region in PET is benign”, GPT-4 provided a detailed list of conditions, including “Symmetry: physiological uptake is often symmetrical”. This suggests that the state-of-the-art LLMs struggle to consistently apply their knowledge to practical contexts.

To address this limitation, we applied prompt engineering to guide LLMs toward producing correct outputs. By framing questions within an explicit context and emphasizing the section of the radiology reports that needs more attention, we were able to improve its accuracy and reliability in interpreting radiology reports. This highlights the importance of supplying domain-specific information into the LLM workflow, ensuring that the model applies clinical logic effectively. Future work will focus on enhancing the reasoning capabilities to further minimize errors and improve trustworthiness of the LLM in radiology report reading. Our study found that Llama-3 is inferior to the GPT-4 model in terms of tumor information extraction. Despite the promising improvements in auto-segmentation precision using LLM-based interpretation of radiologist reports, LLMs are prone to hallucinations. Therefore, continuous validation and human oversight are essential to establish robust guardrails prior to clinical integration.

Radiology reports often specify only the general anatomical region of the tumor and involved nodes, relying on the audience (typically other physicians) having a detailed understanding of human anatomy. While this shorthand is effective for human interpretation, it poses challenges for automated systems such as our FP ruling-out program, which was not trained to understand those terms. To bridge this gap, we defined bounding boxes based on the anatomical structures supported by our DLAS software. While these bounding boxes provide a simplified and somewhat crude representation of anatomical regions, they proved sufficient in this study. However, the limitations of this crude definition are evident. The bounding boxes may not fully capture the complexity and variability of anatomical regions. It is challenging when the tumor is located near the edge of the bounding box. Future work should focus on refining these definitions by leveraging advanced anatomical modeling, integrating probabilistic spatial maps, or employing more sophisticated AI-based methods for a more precise anatomical localization. The improvement will not only benefit the FP ruling-out program but could also benefit other medical image analysis applications.

The contouring agreement, as reflected by the DSC and HD95 metrics, remained inferior to the performance reported in the HECKTOR challenge, even after applying the FP ruling-out approach. This discrepancy was likely due to practice variation in contouring GTVs from PET-CT scans. The contouring practices vary widely, ranging from simple threshold-based methods, such as fixed and adaptive threshold methods [37,38], to more advanced approaches like those based on adaptive region growing [39] and statistical approaches [40]. The PET SUV values vary significantly across patients and scans due to biological process variability, making standardized contouring challenging. Furthermore, PET imaging suffers from a low spatial resolution, a limitation caused by the detector size and the range of positrons before annihilation. Tumor heterogeneity, including necrotic or hypoxic regions, further complicates the delineation of gross tumor boundaries. Consequently, PET signals alone are insufficient for precise GTV contouring, and physicians typically rely on CT anatomical information to supplement the PET data. The extent of contouring beyond the PET-avid region is highly dependent on the physician’s specialty and individual practice [41]. For example, the DSC_mean_ varies considerably among RadOncs (0.56), head and neck surgeons (0.71), and radiologists (0.33). Similar observations were reported in a separate study [42]. The DSCs achieved in this study surpassed the interobserver variability reported in the literature. This suggests that while there is room for improvement such as exploring state-of-the-art neural networks, the proposed approach demonstrates promising consistency and potential in serving as a starting point for reducing GTV contouring variability in HN clinical practice. Additionally, the testing data included 44 institutional cases, which was sufficient to demonstrate the feasibility of utilizing LLMs to improve segmentation precision; however, larger datasets should be explored for future clinical applications.

## 5. Conclusions

DLAS demonstrates significant potential for primary tumor and lymph node segmentation following the false-positive ruling-out process. After applying our approach, the precision of automated segmentation reached 100% for both primary tumors and lymph nodes. The DLAS accurately identified the correct tumor locations and detected all false-negative errors, providing a reliable foundation for radiation oncologists to make the necessary edits. Our developed workflow substantially enhances the accuracy of DLAS in analyzing PET/CT images for head and neck cancer patients. By streamlining the segmentation process, it reduces the manual contouring workload for radiation oncologists, ultimately improving the diagnosis efficiency and supporting a more consistent treatment planning.

## Figures and Tables

**Table 1:** False-positive and false-negative errors for the 44 testing cases before and after the ruling-out process.

Metric	GTVn		GTVp		Overall	
	Before	After	Before	After	Before	After
FP error (count)	17/111	0/111	5/40	0/40	22/151	0/151
FN error (count)	8/111	8/111	0/40	0/40	8/151	8/151
Precision	0.83	1.00	0.88	1.00	0.85	1.00

## Data Availability

Research data are stored in an institutional repository and will be shared upon request to the corresponding author.

## Supplementary Materials

The following supporting information can be downloaded at: https://www.mdpi.com/article/10.3390/cancers17121935/s1. Figure S1: An example of clinical diagnosis report with highlighted colors showing key descriptors of tumors (yellow: key descriptors of tumor, gray: slice location, and pink: non-cancerous descriptor), (b) extraction details for each tumor with GPT using prompt1 and (c) using prompt2; Table S1: Selected OARs for creating bounding boxes of anatomic regions; Table S2: SUVbw statistics of PET images for the GTVn FN error.
