# Supplementary material for: Improving the Precision of Deep-Learning-Based Head and Neck Target Auto-Segmentation by Leveraging Radiology Reports Using a Large Language Model
Source: Cancers (Basel). 2025 Jun 10;17(12):1935. doi: 10.3390/cancers17121935 (PMC12191202; doi:10.3390/cancers17121935)
Supplement: Supplementary file 1 [file cancers-17-01935-s001.zip › cancers-3619274-supplementary.pdf]

(a)

FINDINGS: Liver Max SUV: 3.5
Mediastinum Blood Pool Max SUV: 2.3
HEAD/NECK: The brain parenchyma is unremarkable.
Left tongue base large rim hypermetabolic mass lesion with central photopenic necrosis, measuring around 5.5 x 4.4 cm, max SUV 11.2.
Left neck level 2 hypermetabolic lymph node measuring 2.1 x 2.5 cm with max SUV 14.3.
Left neck level 2B subcentimeter lymph node with max SUV 5.5 on image #83.
Left neck level 3B lymph node measuring 0.9 cm on image #98 with max SUV 10.9.
Right thyroid gland low density nodule with max SUV 2.5.
CHEST: There are no hypermetabolic pulmonary parenchymal lesions. There is no hypermetabolic axillary, mediastinal, or hilar lymphadenopathy.
Physiologic FDG uptake is seen in the myocardium.
Bilateral breast implants in place with periphery calcification.
ABDOMEN/PELVIS: Physiological FDG uptake is seen throughout the liver, spleen, and bowel/colon.
There is physiologic FDG excretion in the kidneys and bladder. There are no hypermetabolic lesions in the adrenal glands. There is no abdominal, pelvic or inguinal lymphadenopathy.
MUSCULOSKELETAL: Bilateral shoulder joints prominent degenerative change, greater on left than right. Bony hardware at the lumbar spine.
IMPRESSION:
1. Left tongue base large mass lesion with central necrosis and rim hypermetabolism, compatible with recurrent malignancy.
2. Left neck multistation hypermetabolic lymph nodes, concerning for nodal metastasis. No PET evidence of distant metastasis.
3. Right thyroid gland nodule with mild degree tracer uptake, concerning for goiter.

(b) Prompt one

Here is the extracted data presented in a table format based on your specifications:

Slice Number	Tumor Size (cm)	SUV Value	Tumor Anatomic Region	Tumor Type	Primary or Node
N/A	5.5 x 4.4	11.2	Left tongue base	Tumor	Primary
83	<1	5.5	Left neck level 2B	Lymph Node	Node
N/A	2.1 x 2.5	14.3	Left neck level 2	Lymph Node	Node
98	0.9	10.9	Left neck level 3B	Lymph Node	Node
N/A	N/A	2.5	Right thyroid gland	Tumor	Nodule

(c) Prompt two

Here's the table based on the findings and impression paragraphs:

Slice Number	Tumor Size (cm)	SUV Value	Tumor Anatomic Region	Tumor Type	Primary/Node
N/A	5.5 x 4.4	11.2	Left Tongue Base	Mass	Primary
83	<1	5.5	Left Neck Level 2B	Lymph Node	Node
N/A	2.1 x 2.5	14.3	Left Neck Level 2	Lymph Node	Node
98	0.9	10.9	Left Neck Level 3B	Lymph Node	Node
N/A	N/A	2.5	Right Thyroid Gland	Nodule	N/A

Figure S1. (a) An example of clinical diagnosis report with highlighted colors showing key descriptors of tumors (yellow: key descriptors of tumor, gray: slice location, and pink: non-cancerous descriptor), (b) extraction details for each tumor with GPT using prompt1 and (c) using prompt2.

Table S1. Selected OARs for creating bounding boxes of anatomic regions.

Anatomical region	Superior	Inferior	Anterior	Posterior	Left	Right
Right nasopharyngeal	Right optical nerve	PCM_S	Cavity_Oral_NRG	Spinal Cord	Joint_TM_L	Joint_TM_R
Right oropharynx	Cavity_Oral_NRG	PCM_M	Bone_Mandible	Pharynx	Parotid_L	Parotid_R

Left hypopharynx	Bone_Mandible	Larynx	Bone_Mandible	Pharynx	Parotid_L	Parotid_R
Tongue base	Cavity_Oral	Bone_Mandible	Bone_Mandible	Pharynx	Glnd_Submand_L	Glnd_Submand_R
Carotid space	Pituitary	Larynx	Cavity_Oral_NRG	Pharynx	Joint_TM_L	Joint_TM_R

Table S2. SUVbw statistics of PET images for the GTVn FN error.

	Mean	Min	Max	Median	Std	Volume(ml)
Case 1	2.09	1.46	2.55	2.1	0.23	0.9
Case 2	1.82	1.01	2.56	1.85	0.37	0.97
Case 3	1.76	0.96	2.4	1.73	0.29	0.71
Case 4	1.35	0.73	2.02	1.35	0.35	0.84
	1.07	0.75	1.52	1.04	0.17	0.35
	1.71	0.91	2.6	1.65	0.41	1.45
Case 5	1.64	1.03	2.37	1.63	0.26	1.76
Case 6	0.97	0.72	1.37	0.97	0.16	0.42
